# Supplementary material for: Genome-wide SNP genotyping as a simple and practical tool to accelerate the development of inbred lines in outbred tree species: An example in cacao (Theobroma cacao L.)
Source: PLoS One. 2022 Oct 26;17(10):e0270437. doi: 10.1371/journal.pone.0270437 (PMC9604995; doi:10.1371/journal.pone.0270437)
Supplement: S3 File — (PDF) [file pone.0270437.s003.pdf]

Supporting information S3 File to:

Uilson Vanderlei Lopes et al. 2022. Genome-wide SNP genotyping as a simple and practical tool to accelerate the development of inbred lines in outbred tree species: an example in cacao (Theobroma cacao L.)

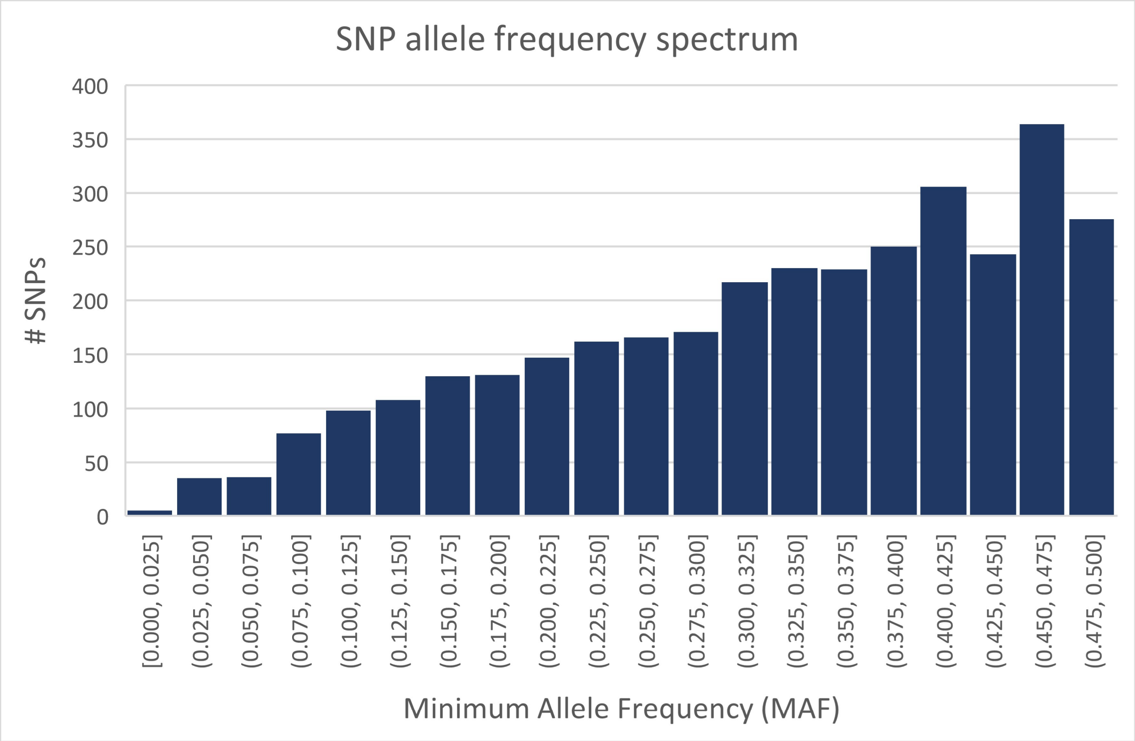

Supporting File S3. Site frequency spectrum of the 3380 SNPs in the 90 *Theobroma cacao* clones studied.
