# Supplementary material for: Genome-wide SNP genotyping as a simple and practical tool to accelerate the development of inbred lines in outbred tree species: An example in cacao (Theobroma cacao L.)
Source: PLoS One. 2022 Oct 26;17(10):e0270437. doi: 10.1371/journal.pone.0270437 (PMC9604995; doi:10.1371/journal.pone.0270437)
Supplement: S6 File — (A) Dendrogram for the 90 clones only and (B) dendrogram for all 139 plants (90 clones and 49 inbred individuals) based on a matrix of genetic distances estimated with 3,380 SNPs. (PDF) [file pone.0270437.s006.pdf]

Supporting information S6 File to: Uilson Vanderlei Lopes et al. 2022. Genome-wide SNP genotyping as a simple and practical tool to accelerate the development of inbred lines in outbred tree species: an example in cacao (Theobroma cacao L.)

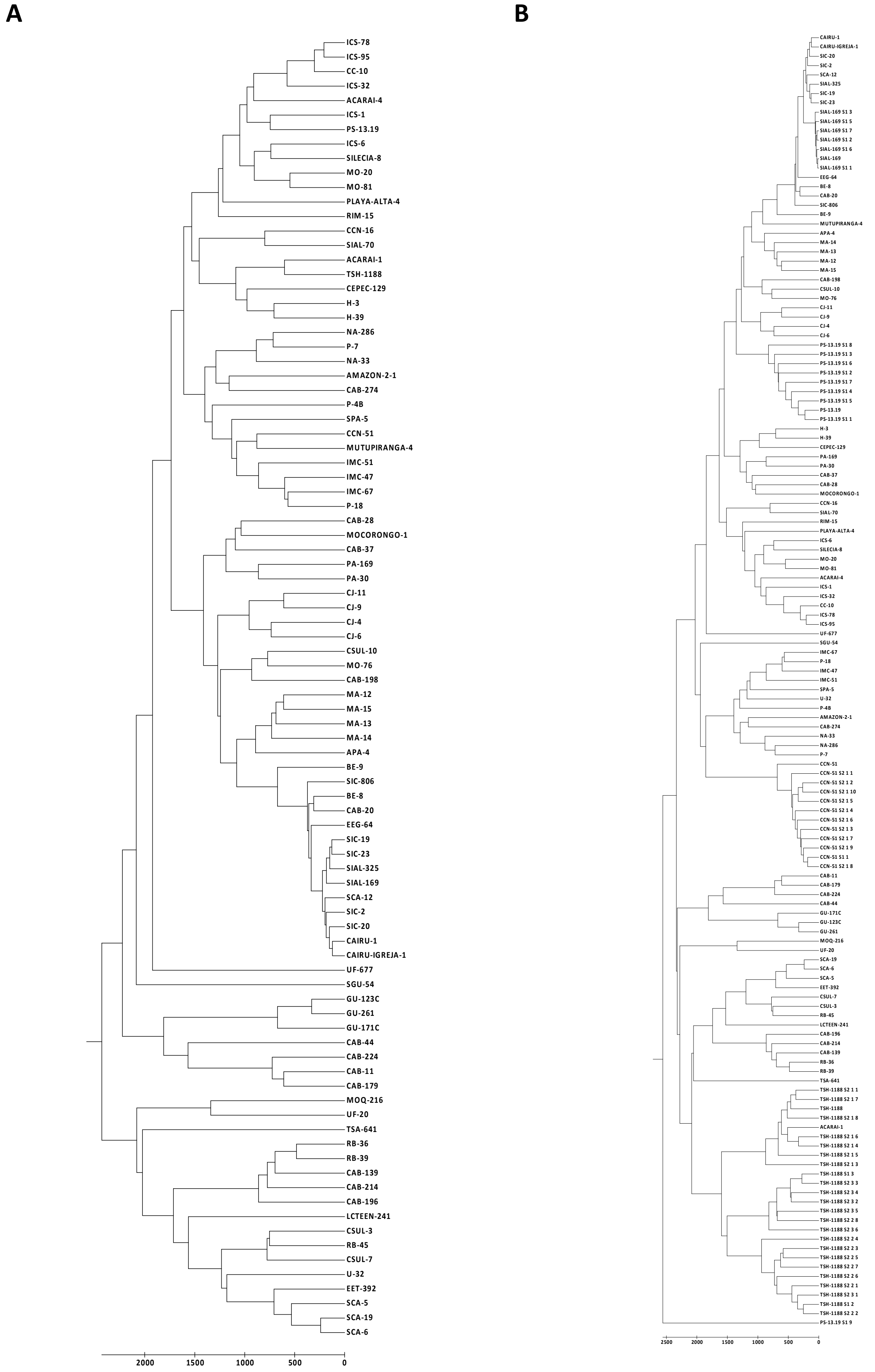

Supporting File S6. UPGMA dendrograms of **(A)** the 90 clones only and **(B)** all 139 plants (90 clones and 49 inbred individuals) based on the matrix of genetic distances estimated with 3380 SNPs.
